# New anticancer therapeutics impact fungal pathobiology, infection dynamics, and outcome

**DOI:** 10.1371/journal.ppat.1011845

**Published:** 2023-12-21

**Authors:** Julia R. Palmucci, Julia A. Messina, Jennifer L. Tenor, John R. Perfect

**Affiliations:** Division of Infectious Diseases, Department of Medicine, Duke University, Durham, North Carolina, United States of America; Vallabhbhai Patel Chest Institute, INDIA

## Introduction

Fungal infections are significant public health concerns, with an estimated 1 billion cases and 1.6 million deaths per year worldwide [1]. Many life-threatening fungal infections develop in immunocompromised individuals with conditions such as HIV, solid organ transplantation, and cancer. Chemotherapy-induced immune suppression is frequently associated with opportunistic fungal infections, often through drug-induced neutropenia and mucositis [2]. Even new targeted therapeutics, such as Burton’s tyrosine kinase (BTK) inhibitors, have been found to predispose cancer patients to fungal infections. While frustrating to clinicians, researchers can use cases of targeted therapy-associated fungal infections to better understand the complex relationship between the fungal pathogen and human host. Alternately, certain anticancer agents have been found to possess inherent antifungal properties. As eukaryotic diseases, cancer and fungal infections present both challenges and opportunities for novel treatments. Even in an era of targeted and precision medicine, opportunistic fungal infections are likely to remain a major factor affecting patient outcome. There is a continued need to uncover the factors influencing treatment-associated infection susceptibility and to identify potential opportunities for drug repurposing.

## How can targeted therapies be used to understand host–pathogen interactions?

Over the past 20 years, small molecule inhibitor and monoclonal antibody (mAb) therapies have revolutionized cancer treatment. Targeted therapies that act upon a specific receptor or signaling molecule were expected to lead to fewer opportunistic infections because they were considered generally less cytotoxic than broadly active chemotherapies [3]. While many early promises of targeted therapeutics have been fulfilled, opportunistic fungal infections continue to emerge among patients undergoing these treatments. In certain cases, in vitro studies and drug mechanism of action have failed to predict fungal susceptibility [4]. Consequently, in 2022, the European Haematology Association released detailed guidelines on antifungal prophylaxis in acute myeloid leukemia patients receiving novel-targeted therapies [5]. The implementation of these guidelines, despite a scarcity of robust clinical studies, indicates the pressing influence of antifungal-targeted therapeutic interactions on patient care.

Targeted therapies also present an opportunity to better understand the dynamics of pathogenesis in the context of human disease and uncover specific pathways and effectors important for pathogen survival within a human host. Here, we describe examples of small molecule inhibitors whose use has furthered our understanding of invasive fungal infections (IFIs). Additional cases are presented in Table 1.

**Table 1:** Fungal infections associated with select anticancer-targeted therapeutics.

Class	Therapy	FDA Indication [6]	Reported Fungal Infection	Ref.
BTK inhibitor	Ibrutinib	CLL SLL WM	Disseminated aspergillosis Disseminated cryptococcosis PJP Various atypical fungal infections	[7–9]
	Acalabrutinib	CLL MCL SLL	Coccidioidomycosis Invasive candidiasis PJP Disseminated aspergillosis	[10–13]
	Zanubrutinib	CLL MCL Marginal zone lymphoma SLL WM	Fungal encephalitis° Fungal pneumonia° Disseminated aspergillosis Disseminated cryptococcosis Scedosporiosis	[14,15]
Multitargeted tyrosine kinase inhibitor	Midostaurin	Acute myeloid leukemia Mast cell leukemia	Invasive candidiasis Invasive fungal infection° Pulmonary aspergillosis *Saprochaete capitata* infection	[16,17]
PI3K inhibitor	Idelalisib	CLL	Coccidioidomycosis Disseminated cryptococcosis *Lomentospora prolificans* infection PJP Pulmonary aspergillosis	[18–21]
	Copanlisib	FL	PJP Pulmonary aspergillosis	[22,23]
	Duvelisib	CLL FL SLL	Fungal pneumonia° PJP	[24,25]
Anti-CD20 antibody	Rituximab	B cell acute leukemia CLL Non-Hodgkin lymphoma	PJP Pulmonary aspergillosis Pulmonary histoplasmosis	[26,27]
	Obinutuzumab	CLL FL	Invasive candidiasis PJP Talaromycosis	[28]
Anti-CD52 antibody	Alemtuzumab	CLL	Disseminated aspergillosis Invasive candidiasis Mucormycosis Pulmonary, cutaneous, and disseminated cryptococcosis	[29–32]
CAR T-cell therapy	Ciltacabtagene autoleucel^	Multiple myeloma	Fusariosis Invasive fungal sinusitis° Mold° Mucormycosis Pulmonary aspergillosis	[33,34]
Anti-CD30 antibody	Brentuximab vedotin	Classical Hodgkin lymphoma Primary and systemic anaplastic large cell lymphoma	PJP	[35]
Anti-CTLA-4 antibody	Ipilimumab	Colorectal cancer Esophageal cancer Hepatocellular carcinoma Malignant pleural mesothelioma Metastatic melanoma Non-small cell lung cancer Renal cell carcinoma	Dermatophytosis Oral and cutaneous candidiasis PJP Pulmonary aspergillosis	[36,37]

Abbreviations: BTK, Burton’s tyrosine kinase; CLL, chronic lymphocytic leukemia; FL, follicular lymphoma; MCL, mantle cell lymphoma; PI3K, phosphoinositide 3-kinase; PJP, *Pneumocystis jiroveci* pneumonia; SLL, small lymphocytic lymphoma; WM, Waldenström’s macroglobulinemia.

°Unspecified.

^^^Representative of class, comprehensive review available [38].

### BTK inhibitors

BTK is an important facet in B cell receptor signaling. Upon B cell receptor activation, BTK is phosphorylated and leads to B cell proliferation and survival [39]. Inactivation of the human BTK gene causes the inherited immune disorder, X-linked agammaglobulinemia, in which neither mature B lymphocytes nor their immunoglobulins are produced [39]. BTK gained prominence as a therapeutic target after it was discovered to be expressed by many B cell malignancies and possesses an antiapoptotic function [40]. Ibrutinib is a first-in-class irreversible BTK inhibitor used in the treatment of chronic lymphocytic leukemia (CLL) and other B cell malignancies. While ibrutinib has been groundbreaking in treating CLL and other cancers, enthusiasm was stunted by a growing number of reports of opportunistic fungal infections, particularly of the central nervous system, including meningitis and cerebral abscesses [8,9]. Increased rates of IFIs were not anticipated as patients with X-linked agammaglobulinemia do not demonstrate increased fungal susceptibility [41]. However, several potential mechanisms of increased susceptibility to fungal infections have since been described in literature, including impairments in macrophage, neutrophil, and platelet antifungal activity [42–44].

In laboratory murine models, both BTK knockout mice and mice treated with ibrutinib showed increased susceptibility to pulmonary aspergillosis [45,46]. Study of calcineurin inhibitors highlighted the importance of BTK in innate immune response to *Aspergillus fumigatus*. In response to phagocytosis of *A*. *fumigatus* by macrophages, BTK activates the calcineurin-NFAT pathway and potentiates TNF-α signaling [47]. Inhibition of this pathway leads to an impaired chemokine response and insufficient neutrophil recruitment, allowing the pathogen to avoid clearance. Further work has indicated that ibrutinib specifically blocks phosphorylation of BTK that activates the host inflammatory immune response to *A*. *fumigatus* [42]. Ibrutinib has also been found to contribute to aberrant innate immune cell response via the TREM-1 (triggering receptor expressed on myeloid cells) signaling pathway. Ibrutinib inhibits the TREM-1 signal cascade and subsequently abrogates antimicrobial activities, including the respiratory burst response of TREM-1-activated neutrophils [45]. More recently, ibrutinib has highlighted the importance of relatively understudied components of fungal immunity. For instance, platelets have pathogen-detecting capabilities, form granules, and are involved in neutrophil extracellular trap formation [44]. Ibrutinib was found to inhibit platelet activation, granule secretion, and platelet-mediated cell damage against *A*. *fumigatus*.

Second-generation BTK inhibitors acalabrutinib and zanubrutinib have since been granted FDA approval and possess greater binding selectivity and fewer off-target effects compared to ibrutinib. While these drugs do not inhibit the interleukin-2-inducible kinase critical for T cell signaling, disseminated fungal infections have still been reported [10]. This suggests that IFI susceptibility is not exclusively a result of off-target effects on T cell response and indicates a greater role of B cell pathways in antifungal immunity. Acalabrutinib, like ibrutinib, was also found to impair antifungal inflammatory responses like M1 macrophage polarization, TNF-α secretion, and neutrophil activation [48,49]. Cumulatively, these inhibitors have been shown to impair the innate immune response through several mechanisms that may lead to a predisposition to IFIs. However, even precise mechanisms may have complex phenotypes. For instance, we have found an association with ibrutinib therapy and disseminated cryptococcosis in human B cell malignancy patients, but precise studies with either BTK-knockout or ibrutinib-treated mice showed no impact on cryptococcosis [50]. Nevertheless, these observations have contributed to a better understanding of complex host–pathogen dynamics and have led to the development of laboratory models for studying fungal–host interactions.

### PI3K inhibitors

The phosphoinositide 3-kinase (PI3K) enzyme family is essential for a range of cellular processes and is hyperactive in a variety of cancers. Class I PI3K sits downstream of G protein–coupled receptors and is important for cellular proliferation and differentiation. Specifically, the p110δ subunit is highly expressed in many hematopoietic malignancies and has been shown to have antiapoptotic effects [51]. Idelalisib is a first-in-class inhibitor of this PI3Kδ isoform that selectively binds to the enzyme’s ATP pocket and is currently used in treatments for relapsed CLL [51,52]. Unfortunately, idelalisib causes relatively high rates of neutropenia early in treatment [53]. Clinical studies found that rates of *Pneumocystis jirovecii* pneumonia (PJP) were significantly higher during treatment, especially when used in combination with other therapies like anti-CD20 mAbs, and several clinical trials were halted due to PJP incidence [54]. PJP prophylaxis has since become standard for patients on idelalisib. There have also been several notable cases of other IFIs reported, including *Lomentospora prolificans*, disseminated cryptococcosis, pulmonary aspergillosis, and a concurrent infection of PJP and *Coccidioides* [18–21]. However, the mechanism underpinning fungal infection susceptibility is unknown.

Previous work has shown that PI3K activity, especially via the p110δ subunit targeted by idelalisib, is required for natural killer (NK) cell fungicidal activity against *Cryptococcus neoformans* by controlling perforin release [55]. The PI3K signaling cascade has also been implicated in epithelial immune response and dendritic cell reactive oxygen species production in response to *Candida albicans* infections [56,57]. Thus, there remains a need for more in vivo testing to pinpoint specific factors following PI3K inhibitor treatment that contribute to diminished antifungal immunity.

### CD-52 monoclonal antibodies

The anti-CD52 antibody, alemtuzumab, is a common treatment for CLL, lymphoma, and autoimmune disorders. CD-52 is expressed on T cells, B cells, and some monocytes, but it is not expressed on stem cells, providing some level of treatment specificity. While the exact mechanism of action is not known, killing of target cells involves complement-induced apoptosis and/or antibody-induced cytotoxicity, leading to a profound T cell lymphopenia [29].

IFIs like PJP, aspergillosis, and cryptococcosis have been associated with alemtuzumab, likely due to the depletion of T cells involved in antifungal immune responses [29,30]. Interestingly, candidiasis is not common in alemtuzumab patients, likely due to the low risk of neutropenia or damage to epithelial cell integrity [29]. Specific research is still needed to understand the risk factors associated with IFI development during alemtuzumab treatment and after exposure, especially concerning the cellular mechanisms that increase susceptibility to non-candidal fungal infections.

## Drug repurposing: Can we use cancer therapeutics to fight fungal infections?

The significant burden of IFIs on the global healthcare system is exacerbated by limited safe and rapidly effective antifungals. Current IFI treatment regimens primarily involve just 3 classes of antifungals—the azoles, echinocandins, and polyenes, in addition to the pyrimidine analog flucytosine. While azoles are frequently used, well tolerated, and exhibit broad-spectrum activity, they primarily inhibit growth rather than directly killing the pathogen. Drug resistance is also a concern due to the fungistatic nature of azoles and their widespread use in agriculture [58]. Furthermore, azoles can cause significant drug–drug interactions with anticancer therapeutics by inhibiting the drug-metabolizing enzyme CYP3A4 [59]. Echinocandins have a narrower scope of antifungal activity and are not effective against all fungal pathogens, especially molds. Polyenes, such as amphotericin B, are highly potent but cause significant side effects in the patient. While a new class of antifungals has not been approved in over 2 decades, there are currently several first-in-class antifungals in late-stage clinical trials, which present promising advances for tolerability and drug–drug interactions [60]. However, the limitations of current antifungal therapies highlight the need to explore alternate approaches to combat fungal infections, with emphasis on achieving rapid fungicidal activity.

One potential avenue of exploration that has gained traction is drug repurposing. Drug repurposing can substantially reduce research and development costs as the compounds have already undergone vigorous clinical testing to understand tolerability, off-target effects, and pharmacokinetics [61]. Fundamentally, there is a large overlap between the treatment paradigms for cancer and fungal infections. As both diseases are eukaryotic, therapeutics often target cellular machinery that is conserved between disease and patient, dramatically increasing the risk of off-target effects. Both fields have struggled with drugs that tend to slow growth of their target rather than killing it outright. However, the commonalities of eukaryotes indicate a potential opportunity for repurposing of certain drugs, especially when those targets are conserved across kingdoms. Here, we present an overview of adapted therapeutics or therapeutic strategies that show promise in treating IFIs (summarized in Table 2).

**Table 2:** Select immune modulating therapies with reported clinical and laboratory antifungal efficacy.

Class	Therapy	FDA Indication [6]	Antifungal Efficacy	Ref.
Anti-PD-1 antibody	Nivolumab	Classical Hodgkin lymphoma Colorectal cancer Esophageal cancer Gastric cancer Hepatocellular carcinoma Malignant pleural mesothelioma Metastatic melanoma NSCLC Renal cell carcinoma Squamous cell carcinoma of the head & neck Urothelial carcinoma	*Aspergillus* spp.^+^ *A*. *fumigatus* *A*. *flavus*^+^ *C*. *albicans* *C*. *neoformans* *F*. *solani*^+^ *Histoplasma capsulatum* *Lichtheimia ramosa*^+^ *Mucor* spp.^+^ *Rhizopus* spp.^+^ *R*. *arrhizus*	[62–71]
Anti-TIGIT antibody	Tiragolumab	NSCLC*	*C*. *albicans*	[72]
Cox2 inhibitor	Celecoxib^&^	Acute pain Ankylosing spondylitis Juvenile RA RA Osteoarthritis Primary dysmenorrhea	*Blastomyces dermatiditis* *Candida* spp. *Coccidioides immitis* *C*. *neoformans* *Fusarium* spp. *Histoplasma capsulatum* *Lomentospora prolificans* *R*. *oryzae*	[73]
RTK inhibitor	Ponatinib	Acute lymphoblastic leukemia Chronic myeloid leukemia	*C*. *albicans* *C*. *neoformans* *S*. *cerevisiae*	[74]
Immunotherapy	IFN-γ	Chronic granulomatous disease Malignant osteopetrosis	*Aspergillus* spp.^+^ *A*. *flavus*^+^ *A*. *fumigatus*^+^ *Candida* spp.^+^ *Cryptococcus neoformans*^+^ Dematiaceous molds^+^ *Mucor* spp.^++^ *Rhizopus* spp.^+^ *Trichosporon beigelii*^+^	[64,65,70,75–80]

Abbreviations: Cox2, cyclooxygenase 2; NSCLC, non-small cell lung cancer; RA, rheumatoid arthritis, RTK, receptor tyrosine kinase; TIGIT, T cell immunoreceptor with Ig and ITIM domains.

*Breakthrough therapy designation.

^+^Denotes clinical use.

^&^Derivative used in antifungal studies.

The story of flucytosine (5-FC) sets a precedent for adapting antitumor compounds to treat fungal infections. 5-FC, a pyrimidine analog, was originally synthesized in 1957, as an antitumor compound [81]. Cellular metabolism of 5-FC produces a toxic nucleotide that interferes with DNA, RNA, and protein synthesis. 5-FC quickly showed potent antifungal activity, but the rapid development of resistance hindered its use as a monotherapy. Currently, it is used in conjunction with amphotericin B to treat cryptococcal meningitis and *Candida* infections [81]. The success of 5-FC in treating systemic yeast infections highlights the need to broaden therapeutic testing parameters so potential novel treatments in a different class are not overlooked or ignored.

### Nivolumab

Nivolumab is a human immunoglobulin G4 antibody that acts as an immune checkpoint inhibitor by targeting the T cell programmed death receptor 1 (PD-1). Tumor cells produce PD-1 and PD-2 ligands, which reduce T cell functionality and promote antigen tolerance and immune evasion [82]. Nivolumab binds the PD-1 receptor to block ligand binding, which leads to a recapitulation of the T cell immune response and an increase in effector T cell proliferation [82].

In the context of infection, sepsis-induced T cell exhaustion is caused by PD-1 and PD-2 ligand up-regulation [66]. To date, nivolumab has been used to treat 1 patient with fusariosis and several patients with mucormycosis—including 3 patients with mixed or coinfections with other fungal and bacterial pathogens [63–65,68,70]. Combination therapy of nivolumab and interferon gamma (IFN-γ) has been reported to reverse immune suppression during fungal and bacterial sepsis by boosting lymphocyte count, monocyte activation, and CD8+ T cells [65,70]. This treatment approach has gathered enthusiasm following the emergence of Coronavirus Disease 2019 (COVID-19)-associated mucormycosis [64]. No case reports have described negative immune consequences in response to treatment, such as cytokine release syndrome.

Data from murine models of histoplasmosis first uncovered mechanistic interactions between the PD-1 receptor pathway and antifungal immunity and served as proof-of-concept that treatment with an anti-PD-1 antibody may lead to enhanced survival during fungal infections [69]. Further work has shown that candidemia, like bacterial sepsis, appears to suppress immune function through T cell exhaustion [83]. Treatment with anti-PD-1 or anti-PD-1-ligand antibodies lead to improved survival in mouse models of systemic *C*. *albicans* infection [66]. Similarly, anti-PD-1 monoclonal antibody treatment was found to increase survival during murine models of *C*. *neoformans* pulmonary infection, aspergillosis, and mucormycosis [62,67,71]. Taken together, this clinical and early laboratory data indicate that nivolumab, especially in concert with other immune-stimulating therapies like IFN-γ, present a promising avenue for the treatment of intractable mold infections.

### Other targeted therapies

There have been other sporadic reports that some antitumor compounds have inherent antifungal activity or may enhance antifungal drug efficacy. TIGIT (T cell immunoreceptor with Ig and ITIM domains) is an immune checkpoint expressed on NK cells and cytotoxic and regulatory T cells [84]. TIGIT-bound *C*. *albicans* can inhibit NK cell activity and immune recognition, and treatment with an anti-TIGIT antibody improved survival in a candidiasis mouse model [72]. A derivative of the cyclooxygenase 2 inhibitor celecoxib, AR-12, was found to have broad-spectrum antifungal activity. AR-12 synergized with azoles and echinocandins against *Candida* and potentiated the effect of fluconazole against *C*. *neoformans* in a systemic murine model [73]. The receptor tyrosine kinase inhibitor, ponatinib, has broad-spectrum antifungal activity in vitro and suppresses the development of azole resistance in *Candida* through targeting of the proton pump, Pma1 [74]. While these compounds are a long way from use in clinical trials, their activity highlights the discovery potential within the field of cancer therapeutics. However, it will be necessary to develop better evaluation systems beyond ad hoc testing to maximize data generation and scientific benefit.

### Immunotherapy

IFN-γ limits tumor growth and may stimulate antitumor immunity [85]. It has been the topic of multiple clinical trials, both as a monotherapy and more recently as an adjunctive therapy. While there are toxicity concerns, IFN-γ has shown clinical promise for ovarian and bladder cancers [86–88]. IFN-γ has also been pursued as an infectious disease treatment because of its immune-stimulating action. Specifically, IFN-γ has been successfully used in combination with nivolumab as an adjunctive immunotherapy for intractable mold infections, and in patients with a poor prognosis due to an underlying condition such as immunosuppression or severe trauma [64,65,70,75–77]. IFN-γ in the treatment of IFIs has begun to be systematically evaluated. A case series of IFN-γ treatment in combination with standard antifungals was found to reestablish immune function in patients with systemic *Candida* or *Aspergillus* infections and potentially improve outcomes in cryptococcal meningitis [78–80].

These effects have begun to be mechanistically interrogated using animal models. An inability to increase IFN-γ expression was associated with significantly higher mortality, while higher expression of IFN-γ was shown to stimulate a Th-1 antifungal immune response and increase fungal clearance during murine models of aspergillosis, pulmonary mucormycosis, and cryptococcosis, respectively [89–91]. While the intracellular mechanisms of IFN-γ treatment for IFIs remain unclear, it is apparent that the immunotherapy presents an exciting potential as an adjunctive therapy for severe fungal infections.

### Radioimmunotherapy

Targeted radioimmunotherapy (RIT) is a treatment in which lethal doses of radiation are delivered to a specific location via the binding of a radioisotope to an antibody. RIT is most frequently used for hematological malignancies like non-Hodgkin lymphoma due to the specificity of antigen presentation on target cell lines [92]. RIT presents a promising avenue for future therapeutic development for infectious diseases due to the specificity of antigen–antibody interactions and the lethal dose of radiation administered, which prevents the emergence of drug resistance [93].

RIT using a bismuth-213 antibody against *C*. *neoformans* glucuronoxylomannan was found to be superior to the standard treatment, amphotericin B, during early systemic *C*. *neoformans* infection in mice [94]. There is a concerted effort to identify antigen targets shared across fungal pathogens (“pan-antigens”) for the development of multipurpose mAbs for RIT [93]. A mAb targeting β-(1,3)-glucan has shown activity in vitro against *C*. *neoformans*, *C*. *albicans*, and *Blastomyces dermatitidis* [95,96]. Recently, safety studies on the bismuth-213 antibody against β-(1,3)-glucan in dogs reported no negative short- or long-term side effects, and the treatment will now move into trials for use in veterinary medicine [97].

The effect of RIT on IFIs may not result from irradiation, alone. In vitro work showed that low-dose radiation did not inhibit growth of *Candida* or *Cryptococcus* species, but fungal burden was substantially reduced in irradiated mice infected with *Candida* or *Cryptococcus* [98]. Another study found that a bismuth-213 antibody against *C*. *neoformans* glucuronoxylomannan not only promoted apoptosis-like cell death but also enhanced the fungicidal activity of macrophages in vitro [99]. More recently, it has been noted that no patients who received low-dose radiation therapy for COVID-19 developed mucormycosis [100]. While there are no modern reports of RIT against IFIs in the clinic, the increased incidence of mucormycosis during the COVID-19 pandemic has returned focus to this strategy for development moving forward.

## What’s next?

As of July 2023, there are 72 protein kinase inhibitors in Phase III clinical trials [101]. As Chamilos and colleagues highlighted, there is a need to develop systemic analyses of the immunosuppressive potential of small molecule inhibitors in the preclinical research stages [7]. This work could better inform clinicians on potential negative patient outcomes and ultimately benefit the cancer, immunotherapy, and fungal research communities. While targeted therapeutic development presents tremendous opportunity for a variety of diseases including cancer and autoimmune disorders, the rapid development of this treatment strategy ensures that fungal infections will continue to be an important facet in clinical outcomes. Several novel antifungals currently in development including the novel triazole opelconazole and the echinocandin rezafungin show promise for prophylactic use in transplant patients [102]. However, further research is acutely necessary to evaluate these compounds for use in concert with targeted therapeutics. In summation, the development of fungal infections during treatment courses with targeted regimens has the potential to deepen our understanding of fungal immunity, uncover the consequences of direct or off-target effects on both host and pathogen, and provide insight into future treatment and management of fungal infections
